# The promising role of hypoxia-resistant insulin-producing cells in ameliorating diabetes mellitus *in vivo*

**DOI:** 10.2144/fsoa-2022-0005

**Published:** 2022-09-14

**Authors:** Hanaa H Ahmed, Hadeer A Aglan, Hanan H Beherei, Mostafa Mabrouk, Nadia S Mahmoud

**Affiliations:** 1 Hormones Department, Medical Research & Clinical Studies Institute, National Research Centre, Giza, 12622, Egypt; 2 Stem Cells Lab, Center of Excellence for Advanced Sciences, National Research Centre, Giza, 12622, Egypt; 3 Refractories, Ceramics & Building Materials Department, Advanced Materials Technology & Mineral Resources Research Institute, National Research Centre, Giza, 12622, Egypt

**Keywords:** diabetes mellitus, hypoxia, insulin-producing cells, mesenchymal stem cells, nanomaterials

## Abstract

**Aim:**

This study aimed to evaluate the efficacy of hypoxia-persistent insulin-producing cells (IPCs) against diabetes *in vivo*.

**Materials & methods:**

Mesenchymal stem cells (MSCs) differentiation into IPCs in the presence of Se/Ti (III) or CeO_2_ nanomaterials. IPCs were subjected to hypoxia and hypoxia genes were analyzed. PKH-26-labeled IPCs were infused in diabetic rats to evaluate their anti-diabetic potential.

**Results:**

MSCs were differentiated into functional IPCs. IPCs exhibited overexpression of anti-apoptotic genes and down-expression of hypoxia and apoptotic genes. IPCs implantation elicited glucose depletion and elevated insulin, HK and G6PD levels. They provoked *VEGF* and *PDX-1* upregulation and *HIF-1α* and *Caspase-3* down-regulation. IPCs transplantation ameliorated the destabilization of pancreatic tissue architecture.

**Conclusion:**

The chosen nanomaterials were impressive in generating hypoxia-resistant IPCs that could be an inspirational strategy for curing diabetes.

## Plain language summary

Transplantation of cells that can release insulin have been reported as an alternate method to islet transfer for curing diabetes; however, the main difficulty facing the quality of the pancreatic cells is the deficiency of oxygen. Thus, this study was done to discover a new curing method for diabetes by producing cells that can release insulin and could survive under low oxygen circumstances, and assessing their healing ability against diabetes in rats.

## Graphical abstract

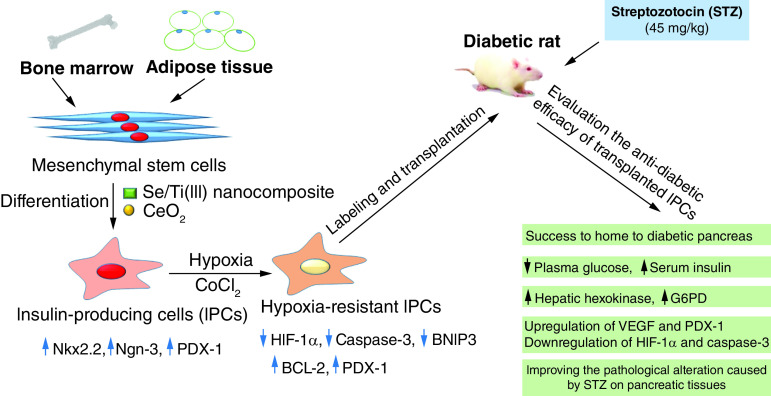

Diabetes mellitus (DM) is characterized by chronic hyperglycemia originating from the disturbance in either insulin release or action or both. Type I DM arises from immune-mediated damage of β-cells in the pancreatic islets, whereas Type II DM, the most frequent form, arises from insulin resistance associated with failure of pancreatic β-cell function [1].

It is estimated that about 463 million individuals, especially those in developing countries, are afflicted with diabetes globally and this number is anticipated to increase to 700 million in 2045. Moreover, diabetes contributes to 1.6 million death cases annually [2]. Diabetes is regarded as an emerging health burden in Egypt since more than eight million people were diagnosed with DM in 2017 [3].

Hyperglycemia is the hallmark symptom of diabetes and long-term hyperglycemia leads to detrimental consequences such as diabetic nephropathy, retinopathy, neuropathy and cardiovascular disease [4]. Moreover, diabetic patients with uncontrolled blood glucose levels are more vulnerable to COVID-19 infection, which increases the rate of mortality, especially during the pandemic [5].

Most of the currently used anti-diabetic medications can modulate the elevated blood glucose level, however, such medications failed to prevent or reverse the disease progression. So, uncover new therapeutic modalities to avoid this caveat is warranted [6].

Many studies shed light on the promising role of stem cells derived from different sources in tissue healing and regeneration [7]. Prior studies by Voltarelli *et al.* [8] and Estrada *et al.* [9] have reported the safety and effectiveness of stem cell-dependent treatment for curing both Type I and Type II diabetes. Mesenchymal stem cells (MSCs) possess many excellent characteristics, including a self-replication capacity, immunomodulatory ability, and multi-lineage differentiation capacity to overwhelm the challenges of ethical concerns, organ availability and allogeneic rejection [10]. This multipotent capacity of MSCs makes them excellent therapeutic agents for curing many ailments, including diabetes mellitus. However, many limitations restrict the utility of MSCs as a therapy for diabetes, such as poor homing and limited differentiation *in vivo* [11].

MSCs originating from various sources were manipulated to differentiate into insulin-producing cells (IPCs) [12]. Insulin-releasing pancreatic beta cells have been reported to be greatly susceptible to hypoxia stress, which is responsible for increased β-cell apoptosis following transplantation [13,14]. It was reported that rat insulinoma cells have survived in the hypoxic environment during xenotransplantation. This could be attributed to their enhanced hypoxia-resistant ability [13].

Selenium (Se) is a coenzyme of many enzymes such as glutathione peroxidase and glutathione reductase. It acts as a free radical scavenger, so it protects against oxidative stress's detrimental impact [15]. In addition, it has been reported to motivate the cell cycle progress and prevent cell apoptosis [16]. In particular, Se nanoparticles exhibit a great biological activity and possess many advantages compared with other nanomaterials owing to their excellent capability to boost immunity and activate the defense response. So, they have been broadly utilized in various medical applications as antioxidant, anticancer, antidiabetic and antimicrobial agents [17].

Titanium dioxide (TiO_2_) belongs to transition metal oxides that possess unique optical, thermal, electric and magnetic characteristics. This oxide performs as a substrate attracting the protein molecules and improving cell attachment. Titanium nanoparticles have been extensively employed in biomedical applications [18] since they were utilized in many dental and orthopedic applications owing to their excellent cell affinity [19].

Cerium oxide (Ce O_2_) is a metal oxide member of the lanthanide group that can exist in both Ce^3+^ and Ce^4+^ ionic states. CeO_2_ nanoparticles have been reported to exhibit a redox activity, free radical scavenging ability, anti-cancer, anti-inflammatory and antibacterial activity [20], which render them to be promising in a range of biomedical applications, including the treatment of diabetes and many neurodegenerative diseases [21].

The principal aim of the present investigation was to explore a novel therapeutic option for ameliorating diabetes mellitus. This goal was attained through: (1) the production of functional insulin-producing cells with enhanced protective ability against hypoxic stress *in vitro*; and (2) evaluation of the therapeutic impact of these cells against diabetes mellitus *in vivo*.

## Materials & methods

### *In vitro* study

#### Nanomaterials

Selenium dioxide/titanium dioxide nanocomposites; Se/Ti (III) (1SeO_2__8TiO_2_) were prepared as previously described in our published paper [22]. Whereas, cerium (IV) oxide (CeO_2_) nanomaterials were obtained from Sigma Company (MO, USA). These nanomaterials were characterized as described in our previous works [22,23].

#### Culture of adipose tissue & bone marrow-derived mesenchymal stem cells

Adipose tissue was isolated from the abdominal and the inguinal fat pad of Wistar rats (8-week old, 120–130 g), supplied from the Animal care facility unit of the National Research Centre, Egypt, after general anesthesia following the method of Tomiyama *et al.* [24]. The adipose tissue was dissected and mixed with phosphate-buffered saline (PBS, Biowest, France). The obtained fat tissue was digested by collagenase Type II (0.075%, Serva Electrophoresis GmbH, Germany) with continuous shaking at 37°C for 1 h. After that, the digested tissue was filtered and centrifuged at 400 × *g* for 10 min at 25°C. Erythrocytes were eliminated by erythrocyte-specific lysis buffer. The isolated cells were then suspended in high glucose Dulbecco's modified Eagle's medium (HG-DMEM, Lonza, Belgium) supplied with fetal bovine serum (30% FBS; Biowest, France) and penicillin-streptomycin (1%, Biowest, France) and incubated at 37°C in a 5% humidified CO_2_ incubator (Sartorius, Germany). After 24 h, any non-attached cells were discarded by replacing the growth medium with a new one. The cells were expanded till obtaining a 90% confluent cell sheet.

Bone marrow content was obtained by flushing the tibiae and femoral bones of 6-week-old Wistar rats (100–120 g) with HG-DMEM supplied with 10% FBS. Cell pellet, obtained after centrifugation, was suspended in a culture medium supplemented with 30% FBS and 1% penicillin-streptomycin. Cells were kept at 37°C in a CO_2_ incubator for 10 days or till achieving large cell clusters [25].

Once bone marrow mesenchymal stem cell (BMSC) and adipose-derived stem cell (ADSC) cultures developed 90% confluence, the cells were subcultured using 1X trypsin/EDTA (Biowest, France) for 5 min at 37°C. Cell passaging was performed till obtaining third passage cultures. MSCs characteristics were identified by flow cytometry screening of MSCs-related markers (CD90 and CD105) and a hematopoietic stem cells marker (CD34) in our previously published paper [22].

#### Conversion of MSCs into insulin-producing cells

ADSCs and BMSCs of third passage were motivated to differentiate into insulin-releasing cells by seeding in HG-DMEM supplied with 5% FBS for 14 days, then cells were suspended in culture media supplied with 10 nmol/l nicotinamide (Bio Basic, Canada) for 7 days, and after that cells were suspended in culture media supplemented with 10 nmol/l nicotinamide and 10 nmol/l exendin-4 (Bio Basic Inc., Canada) [26] along with one of the selected nanoformulations (Se/Ti [III] or CeO_2_) for another 7 days. The concentrations of the tested nanomaterials used for pancreatic differentiation were chosen based on the results of the MTT assay. Concentrations of 5 μg/ml of CeO_2_ and 2 μg/ml of Se/Ti (III) were utilized for ADSCs differentiation. While concentrations of 10 μg/ml of Se/Ti (III) and 20 μg/ml of CeO_2_ were picked out in case of BMSCs differentiation as mentioned in our previous study [22].

#### Characterization of the generated IPCs

##### Quantitative analysis of pancreatic β cells-related gene expression levels

The differentiation of MSCs into pancreatic β-cells was affirmed by estimating the expression patterns of pancreatic β-cell-related genes using real-time PCR. Briefly, total RNA was isolated from both undifferentiated (ADSCs and BMSCs) and differentiated cells (IPCs) using the RNeasy mini kit (cat. #74104, Qiagen, Germany) following the kit's protocol. The isolated RNA (1 μg) was reverse transcribed, following measuring its purity and concentration using NanoDrop 2000 (Thermo Fisher Scientific, USA), using RevertAid cDNA synthesis kit (cat# K1621, Thermo Fisher Scientific, Lithuania) according to the provided manual. The transcriptional patterns of *Nkx2.2*, *Ngn-3* as well as *PDX-1* were assessed by Maxima SYBR Green Master Mix (2X) (cat# K0251, Thermo Fisher Scientific, Lithuania) using QuantStudio 12K Flex real-time PCR system (Applied Biosystems, USA). PCR mixture (25 μl) included Master Mix (12.5 μl), forward primer and reverse primer (1 μl of each), cDNA template (100 ng) and nuclease-free water. The sequences of each primer set are delineated in Table 1. The primer pairs were attained from Invitrogen, USA. A relative comparative method (2^-ΔΔCt^) was utilized to quantify the relative mRNA expression level in differentiated MSCs (IPCs) versus control (undifferentiated MSCs) after being normalized against the *GAPDH* gene [27].

**Table 1.** List of primer sequences of pancreatic β-cell and hypoxia-related genes used in qRT-PCR.

Gene	Primer sequence	Study	Ref.
*Nkx2.2*	F: 5′-CACGCAGGTCAAGATCTG-3′	Wu *et al.*	[26]
	R: 5′-TGCCCGCCTGGAAGGTGGCG-3′		
*Ngn-3*	F:5′-CTTCACAAGAAGTCTGAGAACACCAG-3′		
	R: 5′-CTGCGCATAGCGGACCACAGCTTC-3′		
*PDX-1*	F: 5′-GGTGCCAGAGTTCAGTGCTAA-3′		
	R: 5′-CCAGTCTCGGTTCCATTCG-3′		
*GAPDH*	F: 5′-CACCCTGTTGCTGTAGCCATATTC-3′		
	R: 5′-GACATCAAGAAGGTGGTGAAGCAG-3′		
*HIF-1α*	F: 5′-CACTGCACAGGCCACATTCAT-3′	Yu *et al.*	[28]
	R: 5′-AAGCAGGTCATAGGCGGTTTC-3′		
*Caspase-3*	F: 5′-TGGTACCGATGTCGATGCAGC-3′	Saini *et al.*	[29]
	R: 5′-GGTCCACAGGTCCGTTCGTT-3′		
*BNIP-3*	F: 5′-TCGCTCCCAGACACCACA-3′	Diao *et al.*	[30]
	R: 5′-GCCGACTTGACCAATCCC-3′		
*Bcl-2*	F: 5′-TGACTTCTCTCGTCGCTACC-3′	Thangarajan *et al.*	[31]
	R: 5′-CATCTCCCTGTTGACGCTCT-3′		
*VEGF*	F: 5′-CAGCTATTGCCGTCCAATTGA-3′	Liu *et al.*	[36]
	R: 5′- CCAGGGCTTCATCATTGCA-3′		

#### Establishment of hypoxia stress condition for the generated IPCs

##### Hypoxia induction

The resultant IPCs were exposed to hypoxia by incubating with cobalt (II) chloride (200 μmol/l; Alpha Chemika, India) for 24 h [28].

##### Quantification of hypoxia-specific gene expression

The transcriptional levels of *HIF-1α*, *Caspase-3* and *BNIP-3* along with *Bcl-2* and *PDX-1* were quantified by real-time PCR as previously described in this study. The primer sequences of the hypoxia-related genes are listed in Table 1. A relative comparative method (2^-ΔΔCt^) was utilized to estimate the differential mRNA transcriptional levels of IPCs treated with nanoparticles versus control (IPCs without nanomaterials) after being normalized with the *GAPDH* gene.

#### Cell labeling

The generated IPCs, derived from culturing MSCs in inductive media (IM) supplemented with either Se/Ti (III) or CeO_2_ nanocomposites, were harvested and marked with a PKH26 fluorescent cell linker kit (Sigma, USA) following the associated protocol before their transplantation into the diabetic rats.

The pancreatic tissues of the treated rats were investigated under the fluorescence microscope (Olympus, CKX41, Japan) to confirm the presence of the PKH26-labeled cells.

### *In vivo* study

#### Animals

Male Wistar rats (150–170 g) were placed in a ventilated room with alternative day and night cycles at 25–30°C and provided with water and rat–specific food (Meladco, Egypt). Rats were familiarized with such conditions for 14 days before initiating the experiment.

#### Animal classification

A total number of 48 rats were included in the experiment and separated as follows; negative control group containing 8 rats, which were injected via an intravenous route with sterile saline. While, other rats were subcutaneously injected with only one dose of streptozotocin (45 mg/kg) (STZ, Sigma, USA), after being allowed to fast overnight, for the induction of diabetes mellitus. Streptozotocin was dispersed in sodium citrate buffer (50 mM, pH 4.5) comprising NaCl (150 mM). After 72 h, fasting blood glucose was measured to ensure the induction of diabetes mellitus [32] using the kit purchased from MG Science and Technology Center (Egypt). Rats exhibiting glucose levels greater than 250 mg/dl were considered diabetic and enrolled in the study. After that, the diabetes-induced rats were haphazardly assigned into five groups (8 rats /group). The diabetic group was left without treatment, ADSCs + Se/Ti (III) group which was infused in the tail vein with IPCs (3 × 10^6^/rat) [33], derived from culturing rat ADSCs in inductive media (IM) supplied with 2 μg/ml of Se/Ti (III) nanoformulation, ADSCs + CeO_2_ group which was infused via tail vein with IPCs, generated from culturing rat ADSCs in IM containing 5 μg/ml of CeO_2_ nanoparticles, BMSCs + Se/Ti (III) group which was transfused in the tail vein with IPCs, generated from culturing rat BMSCs in IM supplemented with 10 μg/ml of Se/Ti (III) nanoformulation, and BMSCs + CeO_2_ group which was infused in the tail vein with IPCs, generated from culturing rat BMSCs in IM containing 20 μg/ml of CeO_2_ nanoparticles.

One month later, the food was cut off from the animals for 12 h. After that, blood withdrawal was done, following general anesthesia, from the retro-orbital venous plexus to separate the sera at 1800 × *g* for 15 min at 4°C using a cooling centrifuge for biochemical analyses. Following blood withdrawal, the rats were euthanized and the pancreas and liver of the rats were rapidly isolated and rinsed with ice-cold saline. Liver tissue was homogenized in cold phosphate buffer (50 mM, pH 7.4) to obtain 20% homogenate (W/V) [34]. One portion of each pancreas was immediately preserved at -80°C for the gene expression analysis. Whereas the other part of the pancreas was immersed in 10% formalin for the histological procedure.

#### Assessment of the anti-diabetic efficacy of the transplanted IPCs

##### Biochemical analyses

Serum level of insulin (INS), along with liver hexokinase (HK) and glucose 6-phosphate dehydrogenase (G6PD) activities, were estimated by ELISA kits (Wuhan Fine Biotech Co. LTD, China), following the associated kit's procedure.

##### Molecular genetics study

RNA was extracted from the pancreases of the rats with the aid of Trizol (Invitrogen, USA) and RNeasy mini kit following the method of Carter *et al.* [35]. After confirming the integrity of RNA, cDNA was produced using a cDNA synthesis kit. Gene expression patterns of *VEGF*, *HIF-α*, *PDX-1*, as well as *Caspase-3*, were assessed using a PCR device of DNA-Technology Real-Time (DTlite 4, Russia). The PCR mixture (25 μl) contained QuantiTect SYBR Green master mix (12.5 μl, Qiagen, Germany), 0.8 μl of each sense, and antisense primers of the studied genes (Invitrogen, USA), 100 ng of cDNA and nuclease-free water. Comparative mRNA expression level versus the value of corresponding control was estimated using the 2^-ΔΔCt^ equation following normalization with *GAPDH* [27]. The amplification program included one step of initial denaturation at 94°C for 15 min, succeeded by 40 thermal cycles (94°C for 15s, 60°C for 30 s, 72°C for 30 s). The primer sequences of the studied genes are tabulated in Table 1.

Results were expressed as the fold change in the gene expression pattern of the untreated diabetic group versus the negative control group. While data of all IPCs-treated groups were represented as the fold change in gene expression as compared with the untreated diabetic group.

##### Histological procedure

In brief, after the fixation of pancreas tissues in neutral-buffered formalin (10%) for 24 h, rinsing with water, and dehydration using serially-diluted ethanol were carried out. After that, the tissue samples were embedded in paraffin wax in an oven of 56°C temperature for 6 h. Paraffin-embedded tissue blocks were cut using the microtome. Sections of 5 μm thickness were then placed on glass slides and deparaffinized. Subsequently, they were subjected to staining with hematoxylin and eosin stain to be investigated under the light microscope (Olympus BX51 microscope, Tokyo, Japan) [37].

### Statistical analyses

The current data were displayed as means along with their standard deviation (SD). Data were estimated by the test of one-way analysis of variance (ANOVA) using SPSS 14, succeeded by estimation of the least significant difference (LSD) to compare the significance among different groups. A p-value less than 0.05 was considered significant.

## Results

### Verification of IPCs generation

#### Gene expression analysis of IPCs-related genes

Rat ADSCs incubated in either inductive medium (IM) alone or along with Se/Ti III or CeO_2_ nanomaterials revealed a significant overexpression (p < 0.05) of the *Nkx2.2* gene relative to the undifferentiated ADSCs. Whereas, ADSCs incubated in IM supplied with either Se/Ti III or CeO_2_ nanomaterials displayed a significant upregulation (p < 0.05) of the *Ngn-3* gene as compared with undifferentiated ADSCs and ADSCs incubated in IM alone. Interestingly, ADSCs incubated in IM supplied with Se/Ti III nanomaterials exhibited a significant elevation (p < 0.05) in the *PDX-1* transcriptional level relative to the undifferentiated ADSCs and ADSCs+IM group as well as ADSCs+ CeO_2_ group.

On the other side, rat BMSCs cultured in IM supplied with either Se/Ti III or CeO_2_ nanomaterials showed significant overexpression (p < 0.05) of *Nkx2.2* and *Ngn-3* genes versus undifferentiated BMSCs and BMSCs+IM group. Whereas, BMSCs cultured in either IM alone or supplied with Se/Ti III or CeO_2_ nanomaterials showed a significant overexpression (p < 0.05) of the *PDX-1* gene versus undifferentiated BMSCs. Surprisingly, BMSCs incubated in IM supplied with Se/Ti III nanomaterials displayed a significant overexpression (p < 0.05) of the *PDX-1* gene versus the ADSCs+ CeO_2_ group.

**Figure 1.**
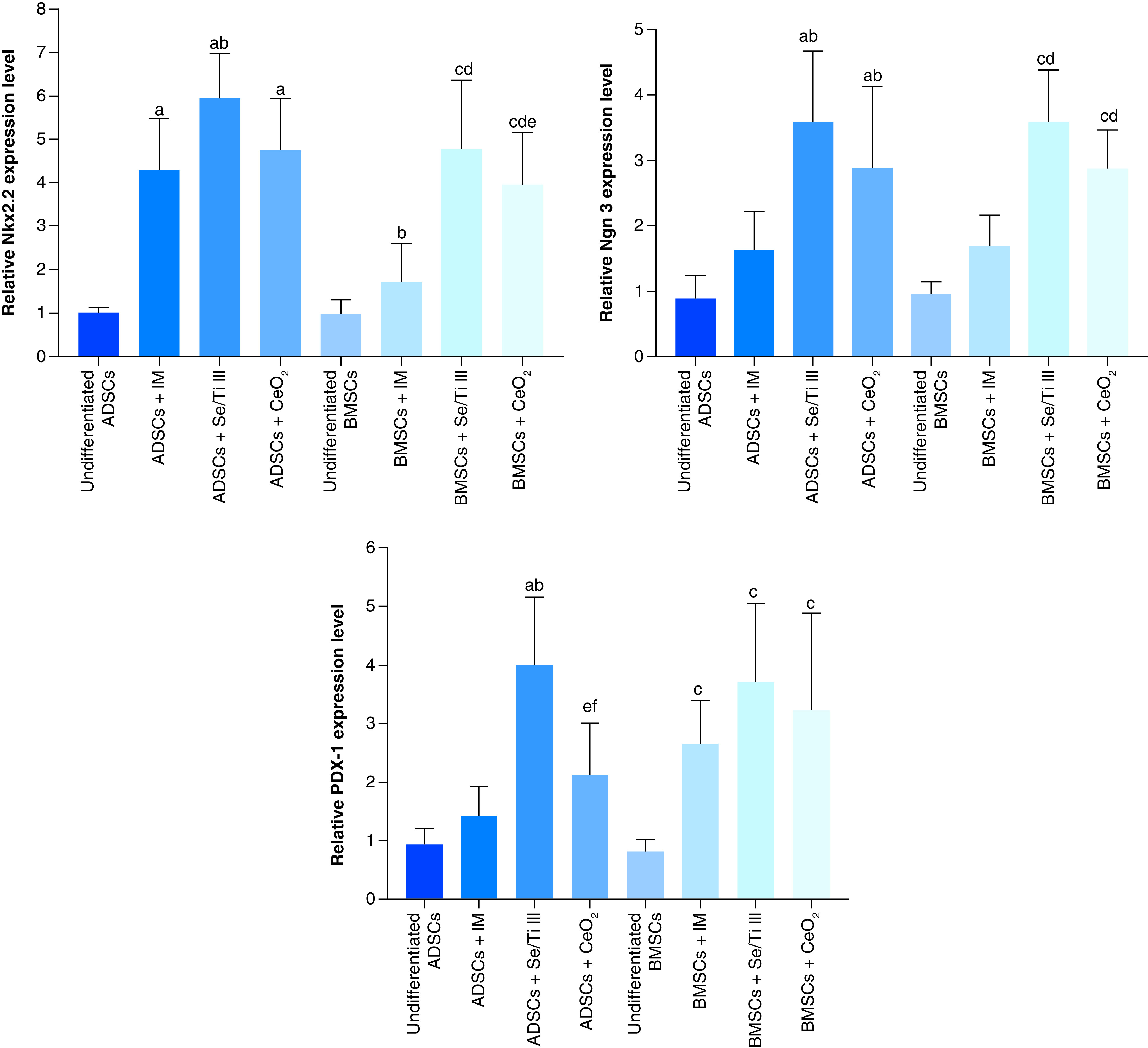
Relative mRNA expression analysis of insulin-producing cells-related genes (*Nkx2.2*, *Ngn-3* and *PDX-1*) in adipose-derived stem cells and bone marrow mesenchymal stem cells-derived insulin-producing cells. Data are displayed as (mean ± SD) procured from four independent experiments (n = 4). **(A)** Significant variation at p < 0.05 relative to undifferentiated ADSCs. **(B)** Significant variation at p < 0.05 relative to (ADSCs + IM). **(C)** Significant variation at p < 0.05 relative to undifferentiated BMSCs. **(D)** Significant variation at p < 0.05 versus (BMSCs +IM). **(E)** Significant change at p < 0.05 relative to (ADSCs + Se/Ti III). **(F)** Significant variation at p < 0.05 relative to (BMSCs + Se/Ti III). ADSC: Adipose-derived stem cell; BMSC: Bone marrow mesenchymal stem cell; IM: Inductive media.

The abovementioned findings confirmed the successful differentiation of both ADSCs and BMSCs into functional IPCs as indicated in Figure 1.

### Hypoxia-related gene expression profile of the *in vitro* cultured IPCs

The hypoxia exposed-IPCs (HE-IPCs) derived from ADSCs cultured in IM supplied with the chosen nanomaterials (Se/Ti [III] or CeO_2_) revealed significant down-regulation (p < 0.05) of *HIF-1α*, *Caspase-3* and *BNIP3* genes accompanied with a significant overexpression (p < 0.05) of *Bcl-2* and *PDX-1* genes relative to those derived from ADSCs cultured in IM without nanomaterials.

**Figure 2.**
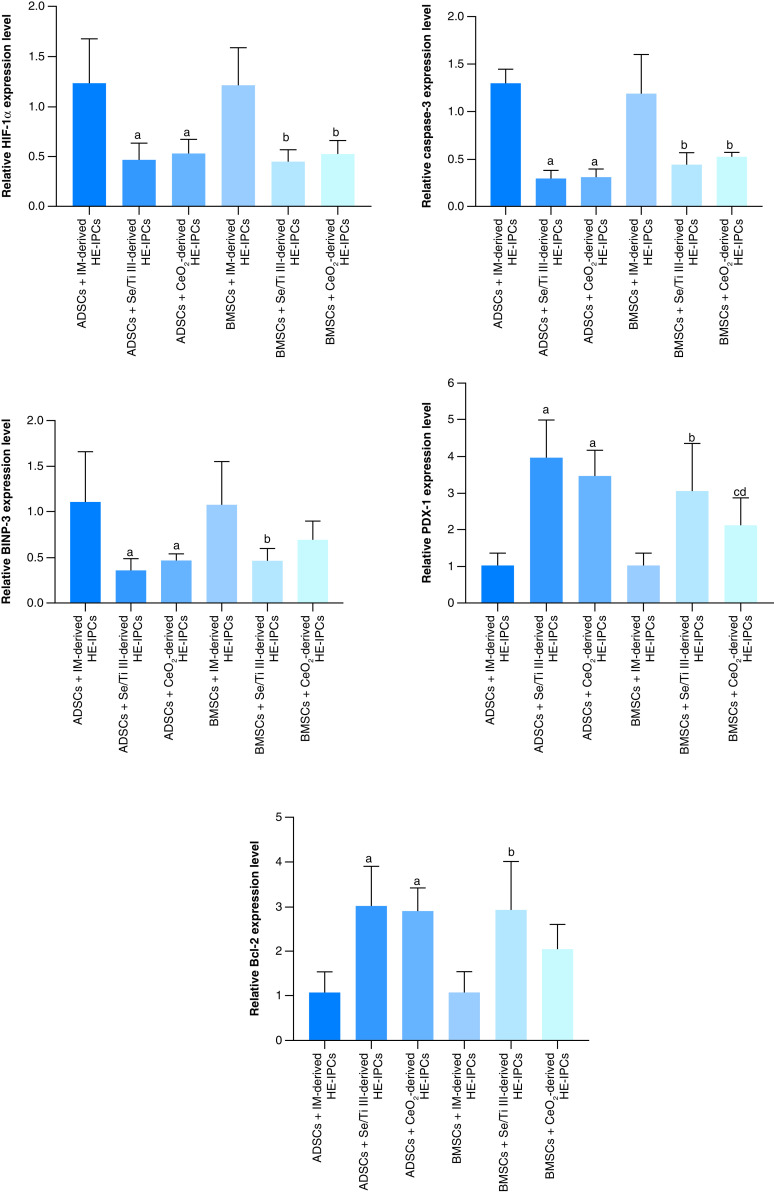
Relative gene expression analysis of hypoxia-related genes; proapoptotic genes (*HIF-1α*, *Caspase-3*, *BNIP3*) and anti-apoptotic genes (*Bcl-2* and *PDX-1*) in adipose-derived stem cells and bone marrow mesenchymal stem cells-derived hypoxia exposed-insulin-producing cells. Data are displayed as (mean ± SD) acquired from four independent experiments (n = 4). **(A)** Significant change at p < 0.05 relative to (ADSCs-derived HE-IPCs + IM). **(B)** Significant change at p < 0.05 relative to (BMSCs-derived HE-IPCs + IM). **(C)** Significant change at p < 0.05 relative to (ADSCs-derived HE-IPCs + Se/Ti III). **(D)** Significant change at p < 0.05 relative to (ADSCs-derived HE-IPCs + CeO_2_). ADSC: Adipose-derived stem cell; BMSC: Bone marrow mesenchymal stem cell; HE-IPC: Hypoxia exposed-insulin-producing cell; IM: Inductive media.

On the other side, HE-IPCs derived from BMSCs incubated in IM supplied with the selected nanomaterials (Se/Ti [III] or CeO_2_) showed significant down-regulation (p < 0.05) of *HIF-1α* and *Caspase-3* genes relative to those derived from BMSCS cultured in IM without nanomaterials. Interestingly, the hypoxia-exposed IPCs generated from BMSCs incubated in IM supplied with Se/Ti (III) nanomaterials displayed significant down-expression (p < 0.05) in the *BNIP3* gene transcriptional level associated with a significant overexpression (p < 0.05) of *Bcl-2* and *PDX-1* genes relative to those derived from BMSCs cultured in IM without nanomaterials Figure 2.

These findings affirm the protective role exerted by the tested nanomaterials on the generated IPCs against the hypoxic stress.

### Homing of the transplanted IPCs

**Figure 3.**
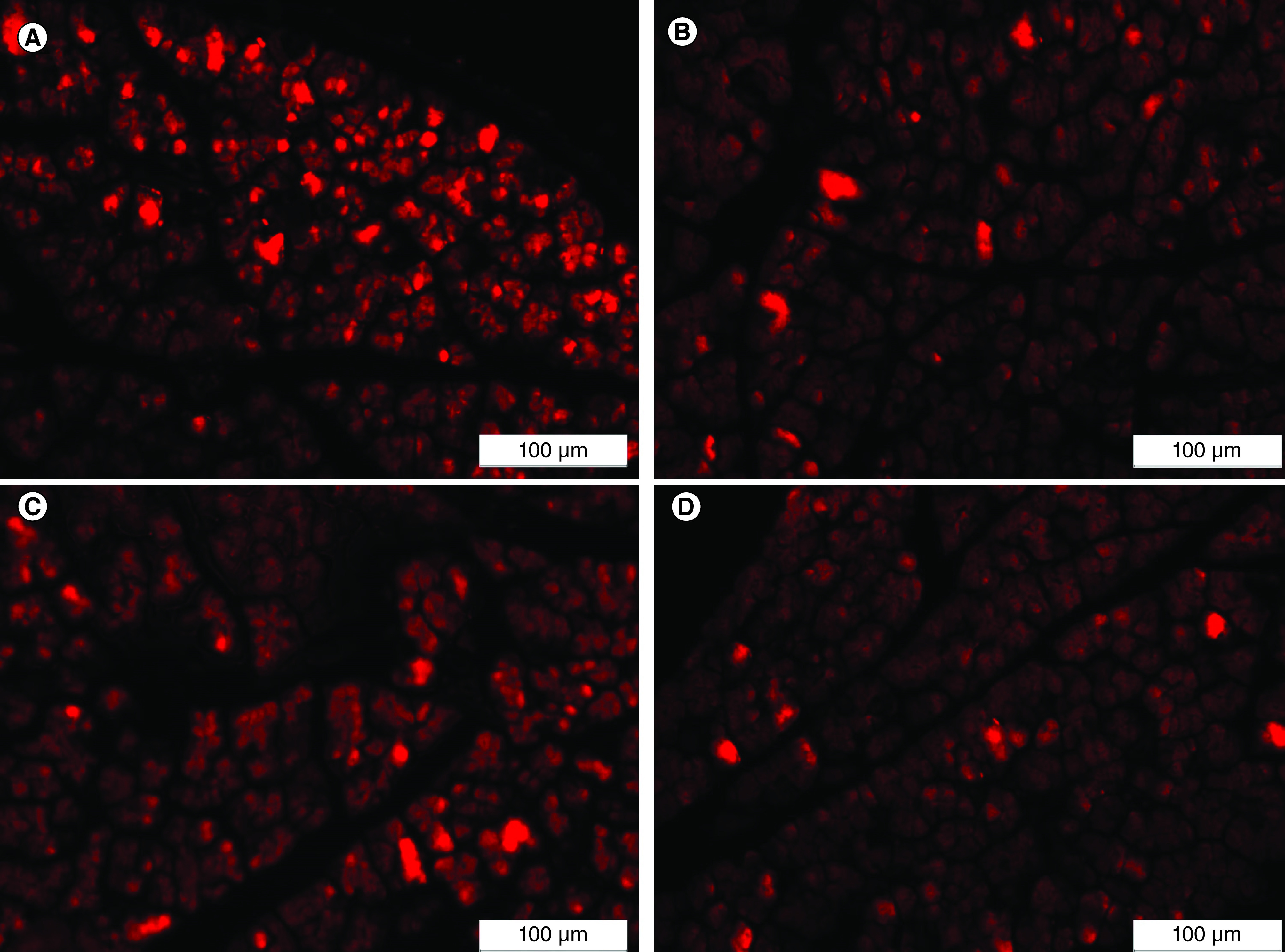
Homing of the implanted insulin-producing cells to the pancreatic tissues of different treated groups indicating the presence of red-labeled cells. **(A)** Adipose-derived stem cells + Se/Ti (III). **(B)** Adipose-derived stem cells + CeO_2_. **(C)** Bone marrow mesenchymal stem cells + Se/Ti (III). **(D)** Bone marrow mesenchymal stem cells + CeO_2_ group.

The homing of the implanted IPCs was confirmed by detecting the presence of PKH26-labeled IPCs in the pancreatic tissue of the treated rats. PKH26-labeled cells were detected in the pancreatic tissue specimens of all IPCs-infused groups (ADSCs + Se/Ti [III], ADSCs + CeO_2_, BMSCs + Se/Ti [III] and BMSCs+CeO_2_ groups) upon examination through the inverted fluorescent microscope which proves the successful homing of the transplanted IPCs to pancreases of the diabetic rats Figure 3.

### Impact of the transplanted hypoxia-resistant IPCs on glycemia

**Figure 4.**
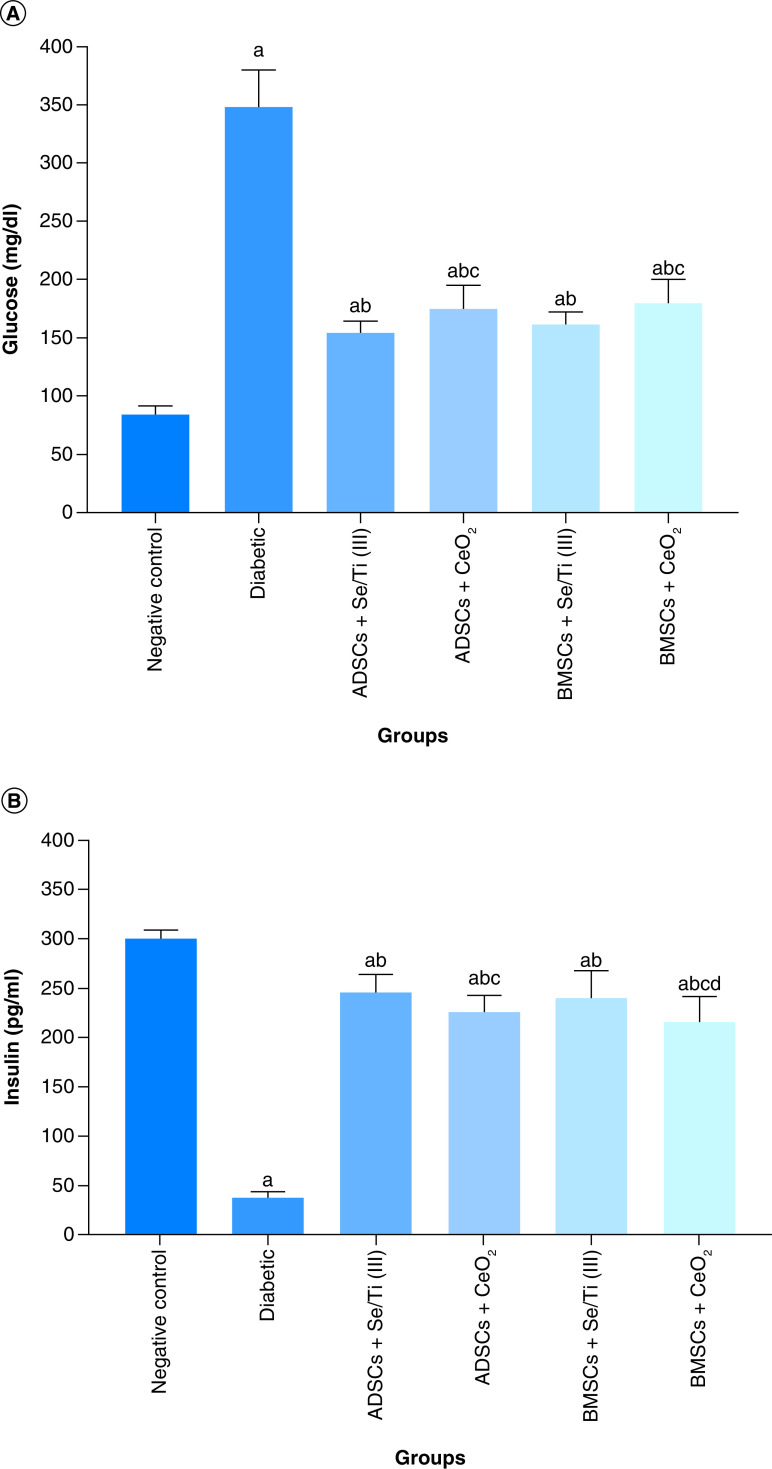
Effect of hypoxia-resistant insulin-producing cells transplantation on plasma glucose and serum level. **(A)** Plasma glucose level of diabetic rats. **(B)** Serum level of insulin of diabetic rats. Values are expressed as (means ± SD) of eight rats per group. **(a)** Significant difference at p < 0.05 when compared with the negative control group. **(b)** Significant difference at p < 0.05 when compared with the diabetic group. **(c)** Significant difference at p < 0.05 when compared with the ADSCs + Se/Ti (III) group. **(d)** Significant difference at p < 0.05 when compared with the BMSCs + Se/Ti (III) group. ADSC: Adipose-derived stem cell; BMSC: Bone marrow mesenchymal stem cell.

Figure 4 represents the impact of the transplantation of the hypoxia-resistant IPCs on glycemia indicators (plasma glucose and insulin serum levels) of diabetes-induced rats. The untreated diabetic rats revealed a significant elevation (p < 0.05) in the blood glucose level along with a significant decrease (p < 0.05) in the serum level of insulin relative to the negative control group. Whereas, implantation of the IPCs evolved from culturing of ADSCs or BMSCs in inductive media containing either Se/Ti (III) or CeO_2_ nanoparticles, in the diabetes-afflicted rats significantly decreased the glucose level and enhanced the insulin serum level (p < 0.05) in comparison with the untreated diabetic rats. Noteworthy, implantation with ADSCs + Se/Ti (III)-derived IPCs displayed a significant decline (p < 0.05) in the serum–glucose level along with a significant increase (p < 0.05) in the serum–insulin level in comparison with ADSCs + CeO_2_ and BMSCs + CeO_2_- derived IPCs. Furthermore, the diabetic rats infused with BMSCs+ Se/Ti (III)-derived IPCs exhibited a significant rise (p < 0.05) in the insulin serum level versus those treated with BMSCs + CeO_2_ -derived IPCs.

### The impact of transplanted hypoxia-resistant IPCs on the activities of hepatic carbohydrate metabolizing enzymes

**Figure 5.**
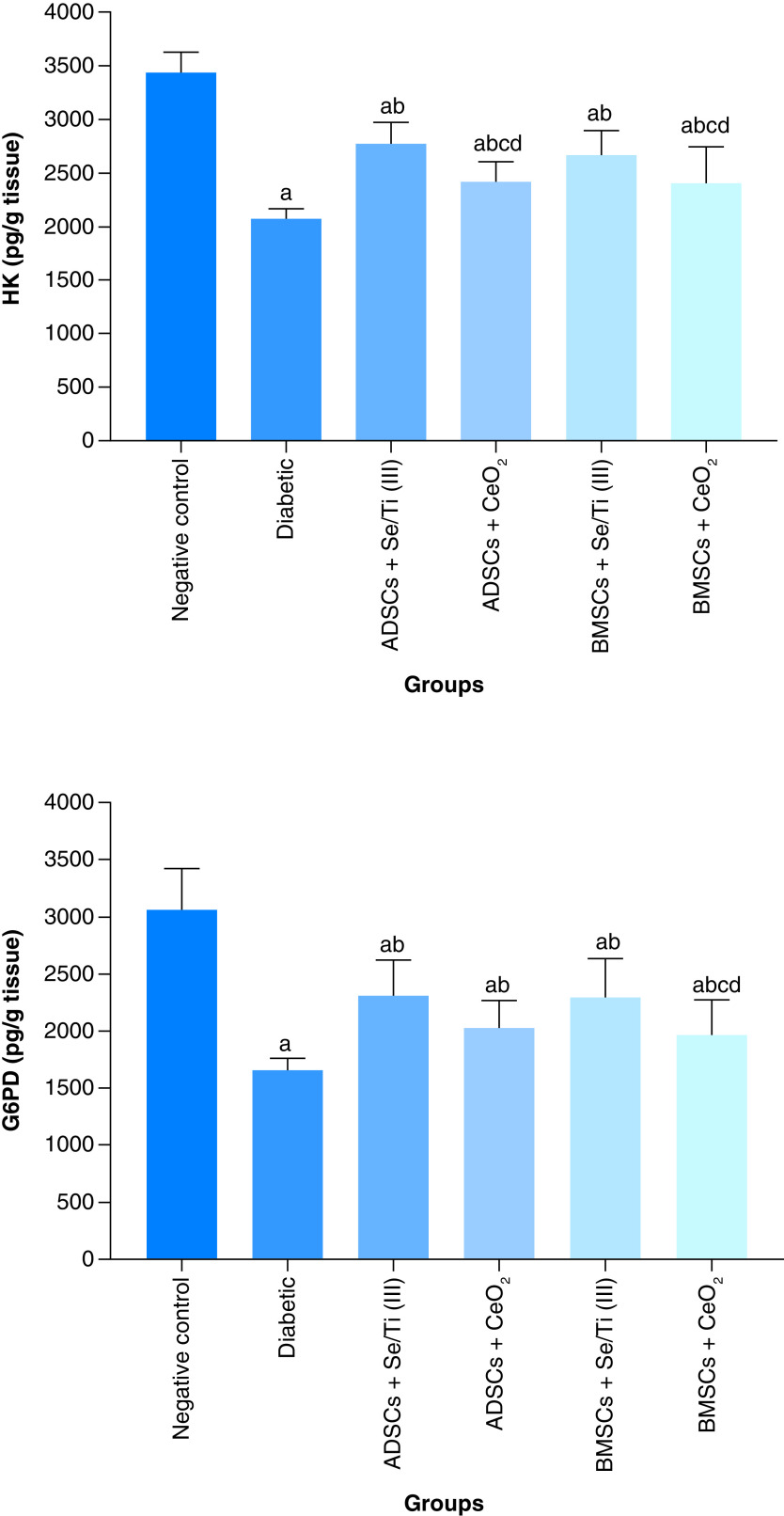
Effect of hypoxia-resistant insulin-producing cells transplantation on the hepatic carbohydrate metabolizing enzyme activities (HK and G6PD) of the diabetes-induced rats. Values are displayed as (means ± SD) of eight rats per group. **(A)** Significant variation at p < 0.05 in comparison with the negative control group. **(B)** Significant variation at p < 0.05 in comparison with the diabetic group. **(C)** Significant variation at p < 0.05 in comparison with the ADSCs + Se/Ti (III) group. **(D)** Significant variation at p < 0.05 in comparison with the BMSCs + Se/Ti (III) group. ADSC: Adipose-derived stem cell; BMSC: Bone marrow mesenchymal stem cell.

Figure 5 displays the influence of transplantation of hypoxia-resistant IPCs on the activity of the hepatic carbohydrate metabolizing enzymes (hexokinase [HK] and glucose 6-phosphate dehydrogenase [G6PD]) of the diabetes-induced rats. The untreated diabetic rats revealed a significant diminution (p < 0.05) in the hepatic HK and G6PD activities as compared with the negative control rats. On the opposite side, all hypoxia-resistant IPCs-infused rats demonstrated a significant rise (p < 0.05) in liver HK and G6PD activities compared with the untreated diabetic group. Moreover, the diabetic rats infused with ADSCs+ Se/Ti (III)-derived IPCs revealed a significant increase (p < 0.05) in HK activity relative to those infused with ADSCs+CeO_2_-derived IPCs or BMSCs+ CeO_2_-derived IPCs. Likewise, BMSCs+ Se/Ti (III) group experienced a significant rise (p < 0.05) in the liver HK activity as compared with ADSCs+CeO_2_ group and BMSCs+ CeO_2_ groups.

Of note, the diabetes-afflicted rats injected with BMSCs + CeO_2_-derived IPCs revealed a significant decline (p < 0.05) in liver G6PD activity relative to those injected with ADSCs + Se/Ti (III)-derived IPCs or BMSCs + Se/Ti (III)-derived IPCs.

### Molecular genetic outcomes

**Figure 6.**
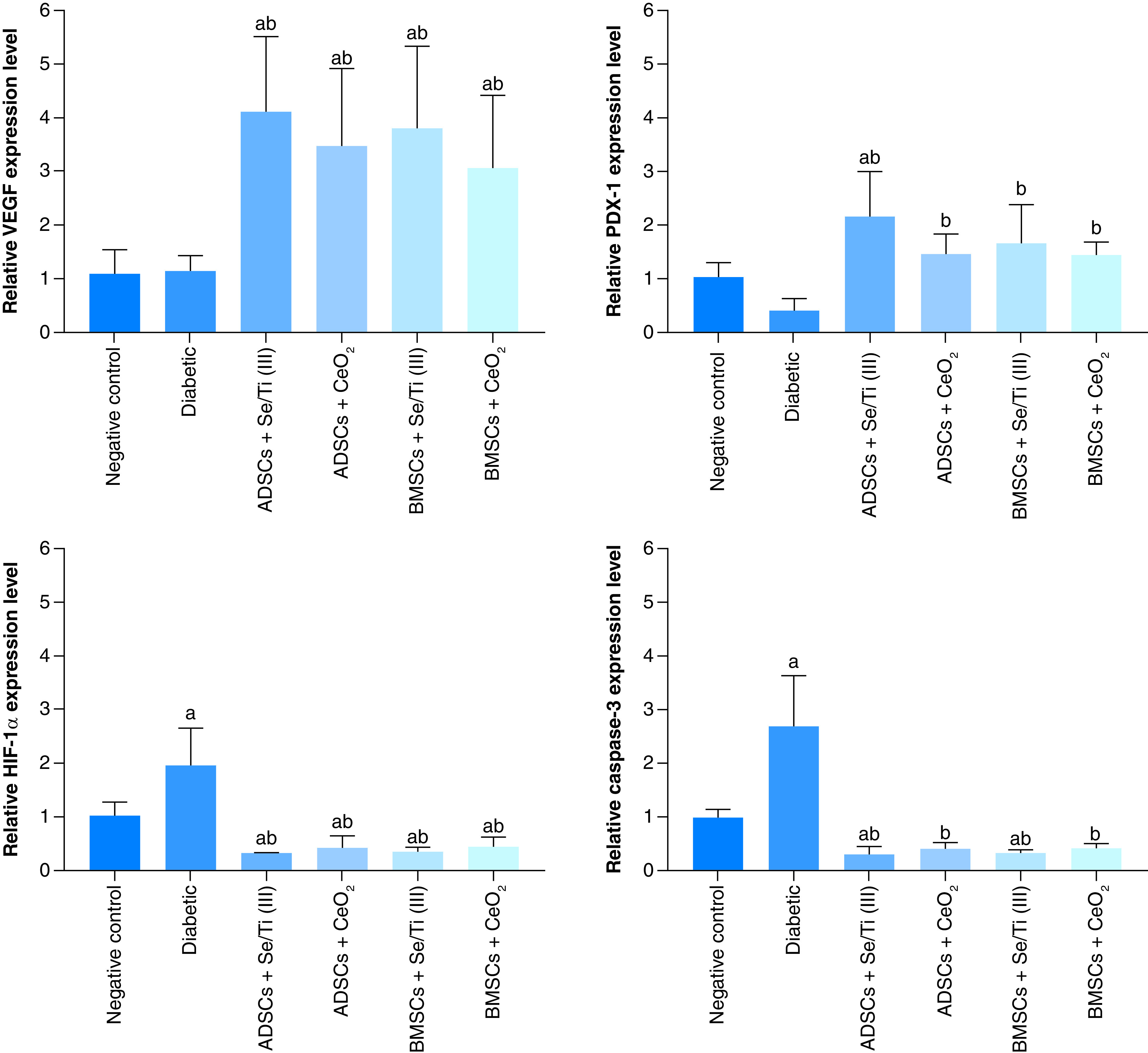
Effect of implanted hypoxia-resistant insulin-producing cells on the pancreatic *VEGF*, *PDX-1*, *HIF-1α* and *Caspase-3* gene expression patterns of the diabetic rats. Values are represented as (means ± SD) of four rats per group. **(A)** Significant variation at p < 0.05 versus the negative control group. **(B)** Significant variation at p < 0.05 versus the diabetic group. ADSC: Adipose-derived stem cell; BMSC: Bone marrow mesenchymal stem cell.

Figure 6 demonstrated the effect of infused hypoxia-resistant IPCs on the expression patterns of pancreatic *VEGF*, *HIF-1α*, *PDX-1* and *Caspase-3* genes of diabetes-induced rats. The untreated diabetic rats displayed insignificant overexpression (p > 0.05) of the pancreatic *VEGF* gene along with insignificant down-expression (p > 0.05) of the *PDX-1* gene. They also experienced a significant upregulation (p < 0.05) of pancreatic *HIF-1α* and *Caspase-3* gene expression patterns compared with the negative control counterparts.

Infusion of the IPCs derived from either ADSCs or BMSCs in the presence of Se/Ti (III) or CeO_2_ nanomaterials in the diabetes-afflicted rats elicited a significant upregulation (p < 0.05) in the pancreatic *VEGF* and *PDX-1* genes in concomitant with a significant down-expression (p < 0.05) of the pancreatic *HIF-1α* and *Caspase -3* genes when compared with the untreated diabetic rats.

### Histological findings

**Figure 7.**
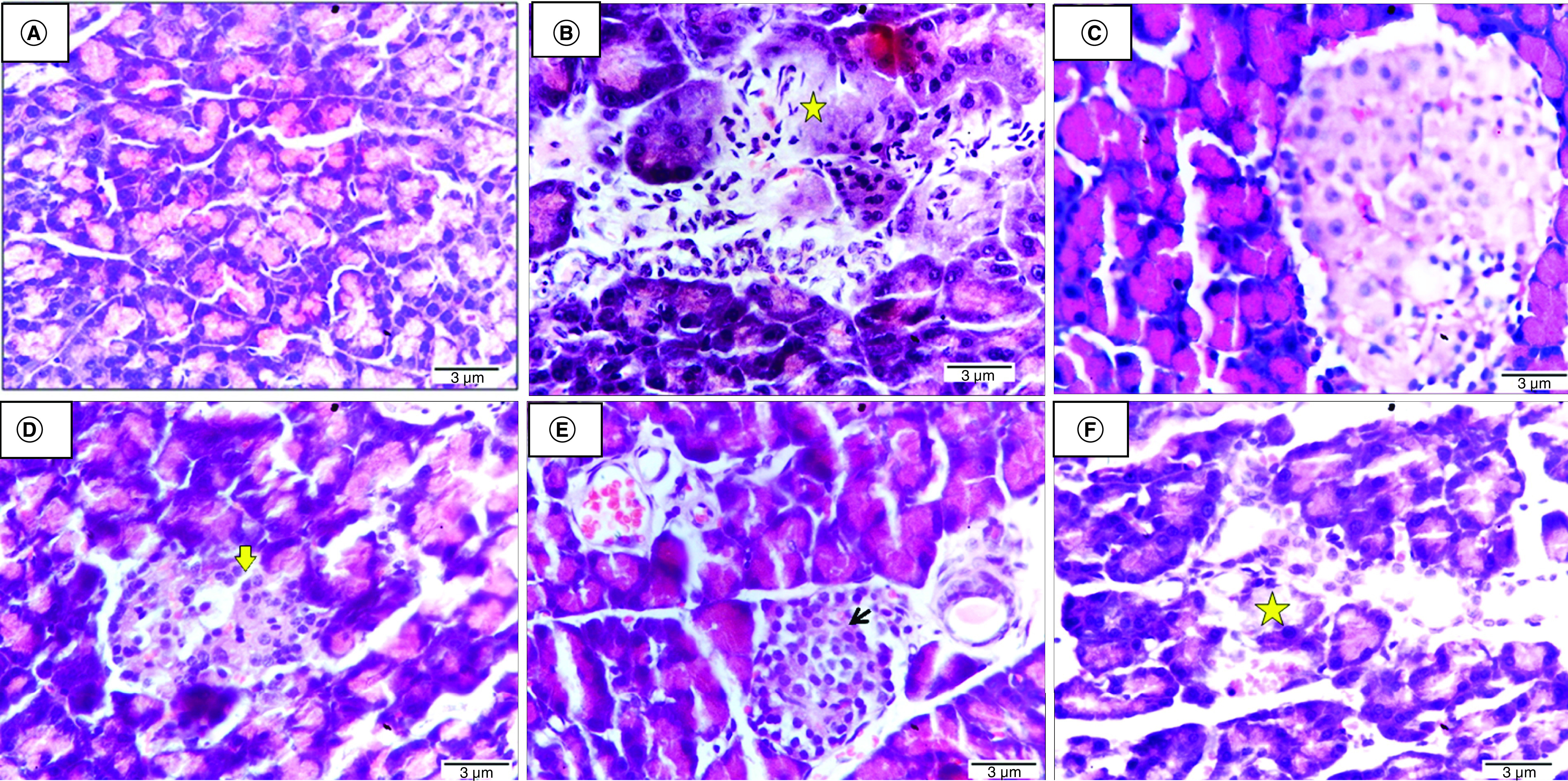
Histological investigations of pancreatic tissue of different groups. **(A)** Negative control rat displaying normal pancreatic architecture with active pancreatic acini. **(B)** Untreated diabetic rat revealing shrinkage of Langerhans islets and pancreatic acini associated with necrosis of components cells (star). **(C)** Diabetic rat infused with ADSCs + Se/Ti (III)-derived insulin-producing cells (IPCs) showing normal Langerhans islets and active pancreatic acini. **(D)** Diabetic rat implanted with adipose-derived stem cells +CeO_2_-derived IPCs showing shrinkage of Langerhans islets and pancreatic acini, with degeneration pyknosis and karyolysis is evident (arrow). **(E)** Diabetic rat infused with BMSCs + Se/Ti (III)-derived IPCs showing normal Langerhans islets (arrow) with some dilatation of blood capillaries and pancreatic ducts. **(F)** Diabetic rat injected with BMSCs + CeO2-derived IPCs showing shrinkage and necrosis of Langerhans islets (star) (H&E, ×400, scale bar: 3 μm).

The histological pancreatic section of the negative control rat displayed normal pancreatic architecture with active pancreatic acini (Figure 7A). While the histological pancreatic section obtained from the untreated diabetic rat revealed shrinkage of Langerhans islets and pancreatic acini, associated with degeneration pyknosis and necrosis of components cells (star) as well as a proliferation of inflammatory cells (Figure 7B). The histological pancreatic section of a diabetes-induced rat injected with ADSCs+ Se/Ti (III)-derived IPCs indicates that Langerhans islets and pancreatic acini are within the normal limit (Figure 7C). Histological pancreatic section of a diabetes-induced rat injected with ADSCs + CeO2-derived IPCs demonstrated shrinkage of Langerhans islets and pancreatic acini, along with degeneration pyknosis and necrosis of components cells, karyolysis is evident (arrow) (Figure 7D). The histological pancreatic section of a diabetes-afflicted rat injected with BMSCs+ Se/Ti (III)-derived IPCs showed normal Langerhans islets (arrow) with some dilatation of the blood capillaries and pancreatic ducts (Figure 7E). A histological pancreatic section attained from a diabetes-induced rat injected with BMSCs + CeO_2_-derived IPCs revealed shrinkage degeneration pyknosis and necrosis of Langerhans islets (star) and pancreatic acini (Figure 7F).

## Discussion

In this study, MSCs originating from rat fat tissue and bone marrow were induced to differentiate into IPCs by an inductive medium containing high glucose concentration, exendin-4 and nicotinamide, which are known as effective inducers for the pancreatic cell differentiation.

Glucose acts as a growth inducer for pancreatic β-cells since it can enhance β-cell proliferation *in vitro* and *in vivo*, enhance the differentiation of adult hepatic stem cells into pancreatic IPCs, and trigger the insulin release from the cells originating from embryonic stem cells. Nicotinamide is a poly (ADP-ribose) synthetase inhibitor that can trigger the differentiation of pancreatic progenitors into IPCs. Whereas exendin-4 can reinforce the proliferation and neogenesis of pancreatic β-cell from ductal progenitor cells, and prevent their death [38].

Many transcriptional factors have been identified to mediate the pancreatic cells' development, differentiation and maturation, including *Nkx2.2, Ngn-3, PAX4, Isl1, PDX1* and *Nkx2.2* is a transcriptional regulator responsible for the differentiation of β-cell lineage and the development of α-cells. Whereas *Ngn-3* is a transcriptional regulator crucial for determining the fate of the pancreatic endocrine progenitor cells. It is known to instigate the transcription of many transcription factors, including *PAX4, NeuroD1, PAX6, Nkx2.2* and *Isl1* which are implicated in the later differentiation of pancreatic endocrine cells [39]. *PDX-1* is a transcriptional factor critical for the development of all types of pancreatic cells and the functionality of mature islet β-cells [40].

The current study revealed the successful generation of IPCs upon culturing ADSCs or BMSCs in inductive media either alone or supplemented with the selected nanomaterials (Se/Ti III or CeO_2_). This was proved by their ability to up-regulate the expression patterns of pancreatic genes; *NKx2.2, Ngn-3* and *PDX-1*. Hashemi Tabar *et al.* [41] reported that IPCs originating from ADSCs exhibited a significant over-expression of *Nkx2.2, Ngn-3* and *PDX-1*. Similarly, the study of Wu *et al.* [26] confirmed the expression of the pancreatic genes, including *PDX-1, Ngn-3* and *NKx2.2* in IPCs differentiated from BMSCs. Moreover, Jafarian *et al.* [42] demonstrated a significant upregulation of *PDX1* and *Ngn-3* and *Nkx2.2* following the pancreatic differentiation of BMSCs.

Some studies demonstrated the difficulty of achieving consistent engraftment of pancreatic progenitor cells [43]. Currently, there are two approaches to avoid hypoxia-induced apoptosis of isolated islet β-cells, either through preconditioning the cells before the transplantation or by oxygen perfusion following engraftment [44]. Thus, the challenge is to determine the best conditions enabling the implanted cells to adapt to hypoxia without alteration in their metabolism.

Exposure to high hypoxia stress activates the expression of *NF-κB*, which in turn induces the apoptotic pathway responses [45]. In addition, hypoxia stimulates the AMP-activated protein kinase pathway, which induces the expression of *Caspase-3*, resulting in β-cell apoptosis [14,46].

In our study, we use cobalt chloride to induce hypoxia *in vitro* to mimic the same conditions facing the transplanted insulin-producing cells and responsible for their poor survival rate upon injection into the circulation which is considered the main obstacles hindering diabetes treatment using IPCs and evaluate whether incubating IPCs with the selected nanomaterials could protect them against the hypoxic injury *in vivo* or not. Cobalt chloride (CoCl_2_) has been reported to induce hypoxia by preventing HIF-1α degradation by replacing Fe^2+^ in the O_2_-binding heme protein; the essential substrate for prolyl hydroxylase by Co^2+^, the enzyme responsible for HIF-1α degradation and consequently stabilizes HIF-α [47]. Hypoxic conditions have been reported to trigger the expression of hypoxia and apoptotic genes, including *HIF-1α, Caspase-3* and *BNIP-3* [48]. Also, hypoxia stress potentiates the down-regulation of anti-apoptotic genes, such as *PDX-1* and *Bcl-2* [49]. Greijer and van der Wall [50] reported that *HIF-1α*, induced in response to low oxygen tension, stimulates apoptosis by interacting with the hypoxia-responsive element in the pro-apoptotic protein, BNIP3, resulting in its activation which in turn triggers the liberation of cytochrome C from mitochondria, resulting in apoptosis [51]. Furthermore, BNIP3 can form a complex with anti-apoptotic protein Bcl-2, yielding its inhibition. This complex activates pro-apoptotic Bax or Bak, resulting in apoptosis [52].

In the current study, we supposed that the addition of nanomaterials to the inductive media of IPCs could enhance the survival rate of these cells against hypoxia. Se is known to stimulate the selenoenzymes and antioxidant enzyme activities, such as glutathione peroxidase (GPx), hence protecting cells from the free radical-induced damage *in vivo* [53]. It also motivates cell cycle progression and inhibits apoptosis [16]. Similarly, CeO_2_ nanoparticles are known as free radical scavengers and thus can protect the cells from the detrimental effect of oxidative injury [54].

The obtained data revealed that the addition of nanomaterials (Se/Ti III or CeO_2_) to the inductive media used for MSCs differentiation into IPCs that were later exposed to hypoxic conditions, increased the expression level of anti-apoptotic genes (*Bcl-2* and *PDX-1*) and decreased the hypoxic and apoptotic genes (*HIF-1α, Caspase-3* and *BNIP-3*) expression patterns of in comparison with those in the absence of Se/Ti III or CeO_2_ nanocomposites. Rao *et al.* [55] mentioned that the functionalized Se nanoparticles prevent oxidative stress by attenuating ROS generation, G2/M phase arrest and intrinsic apoptotic pathway through down-regulating Caspase-3 protein expression in tert-butyl hydroperoxide (t-BOOH)-induced cytotoxicity in PC12 cells. Arya *et al.* [56] cited that CeO_2_ nanoparticles are effective in quenching ROS *in vitro*. Furthermore, Arya *et al.* [57] documented that CeO_2_ nanoparticles inhibited cell apoptosis via maintaining the mitochondrial membrane potential and restoring the NADH/NAD (+) ratio and cellular ATP. Hosseini* et al.* [58] indicated that the isolated rat pancreatic islets cultured in a high glucose-containing medium supplied with CeO_2_ nanoparticles significantly reduced H_2_O_2_-induced apoptosis of islets as verified by the reduced activity of Caspase-3. Moreover, Ghaznavi *et al.* [54] cited that CeO_2_ increased the survival of the undifferentiated PC12 cells subjected to high glucose-triggered oxidative injury and reduced the ROS generation and the down-expression of Caspase-3 protein. Also, these nanoparticles triggered the up-regulated levels of the Bcl-2 protein.

Numerous animal models have been investigated for studying diabetes or evaluating the efficacy of anti-diabetic agents. Streptozotocin (STZ) is regarded as the most commonly recognized diabetogenic drug for creating diabetic animal models [59].

The current investigation revealed the successful homing of the implanted PKH26-stained IPCs derived from MSCs differentiation in the presence of Se/Ti (III) or CeO_2_ nanomaterials to the diabetic pancreas as proved by the detection of PKH26 stained cells in the pancreatic tissue of all IPCs-infused rats. Transplanted stem cells have been reported to successfully accommodate the damaged pancreatic tissues in STZ-induced diabetic animals [33]. Moreover, Ianus *et al.* [60] mentioned that mouse MSCs can differentiate into functional pancreatic β-cells when accommodating to the pancreatic islets following implantation. Nevertheless, the mechanisms by which MSCs are migrated to the tissues and crossed the endothelial cell layer are unknown. The injured tissue possibly expresses particular receptors to enable MSCs adhesion and migration to the injury site. A study performed by Sordi [61] indicated that the MSCs migration to the pancreas tissue could be facilitated by CXCR4–CXCL12 and CX3CR1–CX3CL1 axes, the essential chemotactic regulators known to mediate cell migration.

The data of our work revealed that the untreated diabetic rats exhibited a significant increase in the fasting blood glucose level accompanied by a significant reduction in the insulin serum level indicating the significant clinical signs of diabetes. These findings mirror the previous study of Chen *et al.* [62] and Krishnan *et al.* [63]. STZ is an *N*-nitroso derivative of glucosamine that is exclusively consumed by pancreatic- β-cell via glucose transporter (GLUT-2) causing DNA alkylation. This DNA cleavage results in the activation of poly ADP ribosylation, which in turn leads to a decline in ATP and NAD+ cellular content. Moreover, STZ injection generates superoxide and hydroxyl radicals, which eventually leads to pancreatic β-cells destruction by necrosis [64].

The untreated diabetic rats showed a significant decline in liver HK and G6PD activity contrasted with the negative control group. This result is in coherence with that of Krishnan *et al.* [63]. Our study suggested that the possible reason for the declined activity of the hepatic HK and G6PD could be attributed to insulin depletion following STZ injection. This suggestion is greatly reinforced by the study of Babukumar *et al.* [65]. Many studies have demonstrated that the alteration in glucose metabolizing enzymes is implicated in diabetes pathogenesis and progression [66]. Insulin is known to modulate the activities of many carbohydrate metabolic enzymes such as HK and G6PD [67]. Hexokinase is one of the major glucose metabolic enzymes, that phosphorylates glucose into glucose 6-phosphate. Its inadequacy in diabetes results in reduced glycolysis and decreased consumption of glucose for ATP synthesis. It has been reported that HK is insulin-dependent and has a crucial role in keeping up the glucose homeostasis in all the cells metabolizing glucose by ATP to produce glucose-6-phosphate. While, G6PD catalyzes the initial step in the pentose phosphate pathway, providing NADPH essential for maintaining the reduced glutathione, an important intracellular antioxidant, thus protecting the cells from oxidative damage [65].

According to the present outcomes, untreated diabetic rats exhibited insignificant upregulation in the pancreatic *VEGF* gene expression level concomitant with insignificant down-expression of pancreatic *PDX-1* gene as compared with negative control rats. Furthermore, a significant over-expression of the pancreatic *HIF-1α* and *Caspase-3* genes has been demonstrated in the untreated diabetic rats versus the negative control counterparts. VEGF is a potent mediator of neovascularization, a process of forming new blood vessels from the endothelial cells in the vascular system [68]. VEGF expression is known to be modulated by HIF-1α. Under hypoxic conditions, stabilized HIF-1α undergoes nuclear translocation where it binds to HIF-response elements on the target genes' promoters to activate their transcription with the aid of the co-activator protein p300 [69]. Thangarajah *et al.* [70] reported the aberration of hypoxia-triggered VEGF expression in diabetic tissue *in vitro* as well as in diabetic animals in response to soft tissue ischemia. This could be explained by the reduced functional activity of HIF-1α that modulates the hypoxia-induced *VEGF* expression. These investigators suggested that hyperglycemia mediates the production of superoxide as well as the glycolytic metabolite; methylglyoxal responsible for modification of the co-activator p300 and hence decreases its association with HIF-1α, resulting in impaired *HIF-1α*- mediated *VEGF* gene transactivation and poor vascularization.

On the other side, some studies reported up-regulated VEGF levels in diabetic retinopathy and nephropathy and the vascular dysfunction is mediated by elevated expression of VEGF in the kidney of experimental animals with diabetic complications [71].

*PDX-1* is the main transcriptional regulator responsible for the regulation of pancreatic β-cells differentiation, maturation and function. *PDX-1* is known to regulate the transcriptional activity of *SLC2a2/GLUT2* and glucokinase (*GCK*). Together SLC2a2 and GCK can mediate glucose homeostasis and insulin release [72]. Elevated glucose levels stimulate insulin release by β-cells. Such a process entails glucose uptake by GLUT2 followed by its phosphorylation by GCK [73]. Reduced PDX-1 activity stimulates hyperglycemia and β-cell dysfunction and apoptosis, which is correlated to SLC2a2 and GCK down-expression [74]. SLC2a2 and GCK control the glucose-stimulated insulin release by pancreatic β-cells. Therefore, the downregulated PDX-1 and associated reduced SLC2a2 expression are coupled with hyperglycemia tolerance of β-cells [75].

Our data delineated that diabetic rats experienced a significant upregulation in pancreatic *HIF-1α* and *Caspase-3* gene expression patterns contrasted with the negative control rats. Several investigations have reported that hyperglycemia promotes hypoxic injury and produces mitochondrial ROS [76]. Glomerular mesangial cells in diabetic mice revealed a significant increase in *HIF-1α* expression level. Moreover, Yan *et al.* [77] indicated that the high glucose level induces *HIF-1α* transcriptional activity and promotes *VEGF*; its downstream gene in the endothelial cells. The results of Li *et al.* [78] reported that hyperglycemia could promote hypoxia and stimulate HIF-1α expression in both normal and cancerous pancreas.

Prolonged hypoxia causes the death of pancreatic beta-cells by necrosis [79]. However, activation of apoptotic pathways upon hypoxia exposure has also been reported. Thus, activated Caspase-3 co-localizes with HIF-1α in pancreatic islets, indicating that apoptosis is activated in the islet parts where hypoxia is most profound [50]. The observed upregulation of the pancreatic *Caspase-3* gene in the untreated diabetic rats complies with the results of Chen *et al.* [62]. Hyperglycemia activates the pancreatic RAS, resulting in reduced insulin release and increased β-cell death in diabetes [80]. The enhanced glucose level promotes RAS and activates the transcriptional activity of *AT1R* [62]. Ang II stimulates the generation of ROS via AT1R-coordinated NADPH oxidase and up-regulates the expression of the apoptotic genes, including *Bax* and *Caspase-3* [81].

Histological investigation of the pancreatic tissue section of the untreated diabetic rats showed diffused degeneration and necrosis of Langerhans islets and pancreatic acini associated with infiltration of mononuclear inflammatory cells. These findings are in harmony with those of Krishnan *et al.* [63] who reported shrinkage of islet cells with fatty infiltration upon examination of pancreatic tissue of STZ-induced diabetic rats. Moreover, Chen *et al.* [62] indicated that STZ injection results in swollen islet cells with hyaline degeneration, nuclear dissolution and condensation. Furthermore, Qinna and Badwan [82] demonstrated that diabetic pancreatic sections contain a small number of normal islets and the endochylema of islets displaying granular degeneration and necrosis.

The findings of the current study clarified that the implanted IPCs, originating from culturing MSCs in the presence of Se/Ti (III) or CeO_2_ nanomaterials, significantly reduced the fasting blood glucose level and elevated the serum insulin level in the diabetic rats when compared with the untreated diabetic rats. This comes in line with the study of Hashemi Tabar *et al.* [41] who demonstrated that ADSCs-derived IPCs implantation in STZ-induced diabetic rats could alleviate hyperglycemia and increase insulin secretion. Our study suggested that the observed inhibition of hyperglycemia and elevated serum insulin level following IPCs infusion could be going back to the pancreatic secretion of insulin from the regeneration of Langerhans islets.

A significant rise in hepatic HK and G6PD activity was recorded, following the generated IPCs implantation in the diabetic rats when compared with the untreated diabetic rats. This finding could be explained by the endogenous regenerative activity of the transplanted IPCs in STZ-induced diabetic rats, resulting in elevated insulin secretion and hence stimulating the hepatic carbohydrate metabolizing enzymes' activities [7].

Our data clarified that the infusion of IPCs in the diabetes-induced rats provoked significant over-expression of pancreatic *VEGF* and *PDX-1* genes versus the untreated diabetic rats. The genetic data of Brissova *et al.* [83] indicated that VEGF produced by islets is responsible for revascularization of the transplanted islets. The high islet vascularity is responsible for the quick response to blood glucose and insulin release. Regarding the upregulation of the *PDX-1* gene expression level upon infusion of IPCs in diabetic rats, this result is previously explained by Hashemi Tabar *et al.* [41]. These investigators demonstrated the ability of the implanted cells to express *PDX-1* could be the reason for the observed upregulation in its expression level in pancreatic tissue.

The present findings indicated that the implantation of IPCs in diabetic rats triggered a significant down-expression of the pancreatic *HIF-1α* gene. This observation matches with our *in vitro* outcomes that the generated MSCs–derived IPCs in the presence of nanomaterials showed a significant reduction of *HIF-1α* gene transcriptional level under hypoxic conditions. The observed down-expression of pancreatic *HIF-1α* gene following IPCs transplantation could be attributed to their ability in controlling hyperglycemia as reported in the present investigation, which is the leading factor of hypoxia [78].

Our data clarified that the generated IPCs infusion in diabetes-induced rats elicited a significant down-expression of the pancreatic *Caspase-3* gene which corroborates the finding of Anjum *et al.* [84]. This finding could be allied to the ability of the transplanted cells to manage hyperglycemia and induce insulin release [7]. As it has been demonstrated that high glucose promotes islet cell apoptosis [62].

The histological investigation of the pancreatic tissue sections of IPCs-infused rats revealed that the implanted cells managed to in revert the harmful impact of STZ on the pancreas. The results of Anjum *et al.* [84] study demonstrated that the pre-conditioned IPCs displayed increased survival rate and enhanced angiogenic and pancreatic gene expression levels indicating that these cells may enhance the pancreatic function and architecture. It has been cited that the damaged pancreatic tissues can release large amounts of cytokines and transcription proteins to mediate the repair of pancreatic β-cells or stimulate pancreatic stem cells to produce pancreatic β-cells [85].

## Conclusion

The current investigation clarifies that the tested nanomaterials (Se/Ti III or CeO_2_) can potently promote the conversion of MSCs into insulin-releasing cells and potentiate their ability to survive under low oxygen circumstances *in vitro* and *in vivo*. This pronounced effect could be allied to the ability of these nanomaterials to scavenge free radicals and re-establish the balance between apoptotic and anti-apoptotic genes. Strikingly, our study highlights the anti-diabetic efficacy of the generated IPCs in alleviating the detrimental impact caused by hyperglycemia and hypoinsulinemia. This superior effect could be attributed to their superior capability to accommodate the diabetic pancreas following systemic implantation, control blood glucose, restore the pancreatic insulin synthesis and secretion from the regenerated β-cell regeneration, retrieve hexokinase and glucose-6-phosphate dehydrogenase activities, promote angiogenesis, attenuate hypoxia and inhibit apoptosis. Hence, hypoxia-resistant insulin-producing cells could represent a future promising candidate for ameliorating the pathological indices of diabetes mellitus.

## Future perspective

This approach highlights the impressive role of the chosen nanomaterials in generating hypoxia-resistant IPCs that offer an inspirational strategy for treating diabetes. In the future, hypoxia-combating IPCs could be an alternative to islet transplantation that lacks long-term functionality due to oxygen deprivation following implantation.

**Summary points**

- Mesenchymal stem cells of either adipose tissue or bone-marrow source were successfully differentiated into functional insulin-producing cells (IPCs) as proved by the upregulation of pancreatic β-cell-related gene expression.
- Se/Ti(III) and CeO_2_ nanomaterials were proved to effectively protect the generated insulin-producing cells from hypoxia conditions.
- The generated IPCs were able to combat hypoxia stress *in vitro* as evidenced by the upregulation of anti-apoptotic genes and down-regulation of hypoxia and apoptotic gene expression levels.
- The generated PKH-26-labeled IPCs were successfully home to the diabetic pancreas following transplantation.
- IPCs implantation *in vivo* provoked a significant decline in glucose level as well as a significant increase in insulin serum level, HK and G6PD activities. Also, it could inverse the detrimental impact of streptozotocin on the pancreatic tissue architecture suggesting the anti-diabetic potential of the infused IPCs.
- The infused IPCs could also combat hypoxia *in vivo* as evidenced by the significant upregulation in *VEGF* and *PDX-1* and down-regulation in *HIF-1α* and *Caspase-3* gene expression levels.
- Such prominent effect of hypoxia-resistant IPCs required to be applied in a clinical setting.
